# Effects of p21 on adult hippocampal neuronal development after irradiation

**DOI:** 10.1038/s41420-018-0081-2

**Published:** 2018-07-18

**Authors:** Yu-Qing Li, Chong Shun Wong

**Affiliations:** 1 Sunnybrook Health Sciences Centre, University of Toronto, Toronto, ON Canada; 2 Departments of Radiation Oncology and Medical Biophysics, University of Toronto, Toronto, ON Canada

**Keywords:** Neural stem cells, Cell death in the nervous system

## Abstract

Inhibition of hippocampal neurogenesis is implicated in neurocognitive impairment after cranial irradiation. We recently demonstrated that disruption of neuronal development after DNA damage was regulated by p53. The cyclin-dependent kinase inhibitor 1 or p21, a downstream effector p53, mediates cell cycle arrest in response to DNA damage. There is evidence that p21 negatively regulates proliferation of neural progenitors (NPCs). Here we characterized the effects of p21 on disruption of neuronal development in the hippocampal dentate gyrus after irradiation. We irradiated young adult mice wild type (+/+) or knockout (−/−) of the *Cdkn1a* (*p21)* gene, and used different bromodeoxyuridine (BrdU) paradigms for cell fate mapping. The acute apoptotic response of NPCs in the subgranular zone of the dentate gyrus was independent of p21 after irradiation. In nonirradiated mice, *p21* knockout resulted in an increase in neuroblast proliferation and neurogenesis. At 9 weeks after 5Gy, NPCs in the subgranular zone demonstrated increased p21 expression. Loss of newborn type-1 cells and disruption of hippocampal neurogenesis was evident at 9 weeks after irradiation, and these effects were independent of *p21* genotype status. Within the developmental milestones of NPCs, irradiation resulted in loss of early intermediate NPCs (type-2a cells) in wild-type mice, whereas the principal effect of irradiation with p21 loss was culling of proliferating late intermediate (type-2b cells) and neuroblasts. These results suggest that p21 exerts differential effects on cell fate of NPCs after irradiation. p21 may serve to protect proliferating late NPCs but does not alter the ultimate inhibition of new neuron production after DNA damage.

## Introduction

Multipotent neural stem cells and/or neural progenitor cells (NPCs) are present in the adult mammalian central nervous system. In the adult mammalian brain, the dentate gyrus of the hippocampus represents an area where NPCs continue to generate new neurons which become integrated into the neuronal circuitry^1,2^.

Many physiologic conditions such as an enriched environment and exercise have been reported to result in enhanced adult neurogenesis^3^. Neuronal development in the adult hippocampus is disrupted in various pathologic conditions and brain injuries^1,2^ including after ionizing radiation^4^. Neurogenesis is associated with hippocampal function of learning and memory^5,6^. Inhibition of neurogenesis is implicated in neurocognitive decline following radiation treatment for brain tumors^4^. How DNA damage following ionizing radiation leads to impaired neuronal development in the adult hippocampus remains unclear^7^.

In the commonly accepted model of hippocampal neuronal development, radial glial-like cells or type-1 cells are thought to be the neural stem cells^2^. They give rise to transient amplifying or intermediate NPCs (type-2a, type-2b, and type-3 cells) which differ by their potential for proliferation and increasing neuronal differentiation^8^. NPCs in the adult mouse hippocampus are known to undergo apoptosis after irradiation^9^, a response mediated by the tumor suppressor p53^10,11^. Despite the absence of NPC apoptosis, p53 loss resulted in increased ablation of newborn type-1 cells and profound inhibition of adult neurogenesis after irradiation^12^. Activation of p53 after irradiation results in upregulation of its downstream effector, the cyclin-dependent kinase inhibitor 1 or p21. There is evidence that p21 negatively regulates NPC proliferation^13^. Here we asked whether p21 might play a role in disruption of hippocampal neuronal development after irradiation. Using mice wild type (+/+) or knockout (−/−) of the *p21* gene, p21 was found to have differential effects on cell fate of NPCs, and specifically on disruption of the intermediate NPC stages of neuronal development after irradiation. Loss of p21 however did not alter the extent of inhibition of production of new neurons after irradiation.

## Results

### Apoptosis of neural progenitors after irradiation is independent of p21

**Fig. 1:**
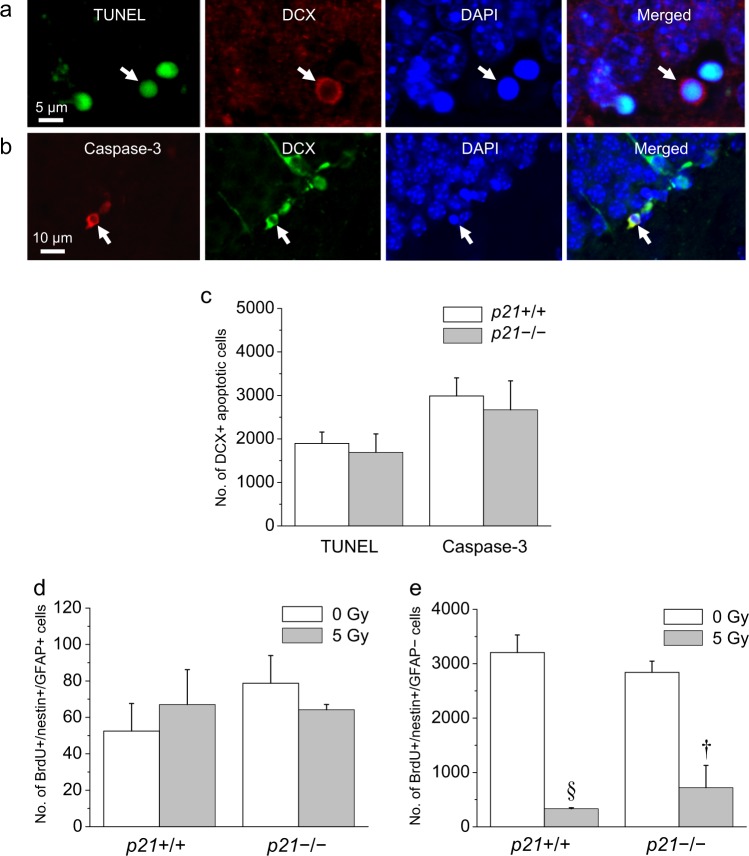
Irradiation induces apoptosis of neuronal progenitors in the subgranular zone of dentate gyrus independent of p21. **a** A TUNEL+ cell (arrow, green) is DCX+ (red, arrow) and demonstrates characteristic nuclear condensation (DAPI, blue). **b** A caspase-3+ cell (arrow, red) shows perinuclear DCX immunoreactivity (green) and nuclear condensation on DAPI (blue). **c** There is no difference in the number of DCX+ apoptotic cells labeled by TUNEL or caspase-3 at 8 h after 5 Gy between wild-type (+/+) mice and *p21*−/− mice. **d** At 24 h after 5 Gy, there is no loss of BrdU+/nestin+/GFAP+ cells in dentate gyrus of *p21*+/+ mice and *p21*−/− mice. **e** A marked reduction in BrdU+/nestin+/GFAP− cells is noted at 24 h after 5 Gy regardless of *p21* genotype. BrdU (50 mg/kg) was given every 2 h for four doses, and animals were irradiated with a single dose of 0 or 5 Gy immediately after the final BrdU injection. Data are represented as mean ± SEM and analyzed using two-way ANOVA, †*p* < 0.01, §*p* < 0.001, post hoc Bonferroni comparison with 3−4 mice per experimental group

Within hours after irradiation, there is a robust p53-mediated apoptotic response of NPCs in the subgranular zone of the dentate gyrus^11^. Two apoptosis radiosensitive NPC subpopulations, proliferating type-2 cells and nonproliferating neuroblasts (type-3 cells) have been described^14^. We first determined whether p21, a downstream effector of p53, plays a role in radiation-induced apoptosis. Using nonbiased stereology, we compared the number of apoptotic cells at 8 h, the peak apoptotic response after 5Gy in the dentate gyrus of *p21*−/− mice with *p21*+/+ mice. Apoptotic cells with characteristic morphology on 4′,6-diamidino-2-phenylindole (DAPI) nuclear staining and positive (+) for terminal deoxynucleotidyl transferase dUTP nick-end labeling (TUNEL) or caspase-3 (Fig. 1a, b) were readily observed in the subgranular zone of both irradiated *p21*+/+ mice and *p21*−/− mice. Using doublecortin (DCX) to label neuroblasts, we observed no difference in the number of TUNEL+/DCX+ and caspase-3+/DCX+ cells in *p21*−/− mice compared to *p21*+/+ mice at 8 h after irradiation (Fig. 1c).

Since only a fraction of apoptotic cells expressed nestin, a marker of early NPCs^14^, we determined loss of proliferating type-2 cells at 24 h to provide evidence for apoptosis in this apoptotic-sensitive NPC subpopulation^14^. Bromodeoxyuridine (BrdU, 50 mg/kg) was given every 2 h for four doses, and animals were given a single dose of 5 Gy immediately after the final BrdU injection. No change in the number of BrdU+/GFAP+/nestin+ cells with characteristic radial glia morphology (type-1 cells) in irradiated *p21*+/+ mice and *p21*−/− mice at 24 h was observed compared to their respective 0-Gy controls (Fig. 1d). A marked loss of BrdU+/nestin+ but GFAP-negative (−) cells or proliferating type-2 cells was observed in irradiated *p21*+/+ mice and *p21*−/− mice after 5 Gy compared to 0-Gy genotype controls (irradiation, *p* = 0.001; *p21* genotype, *p* = 0.8; two-way ANOVA; Fig. 1e). These results are consistent with the lack of a role for p21 in the apoptotic response of NPCs after irradiation.

### Expression of p21 is increased in neural progenitors after irradiation

**Fig. 2:**
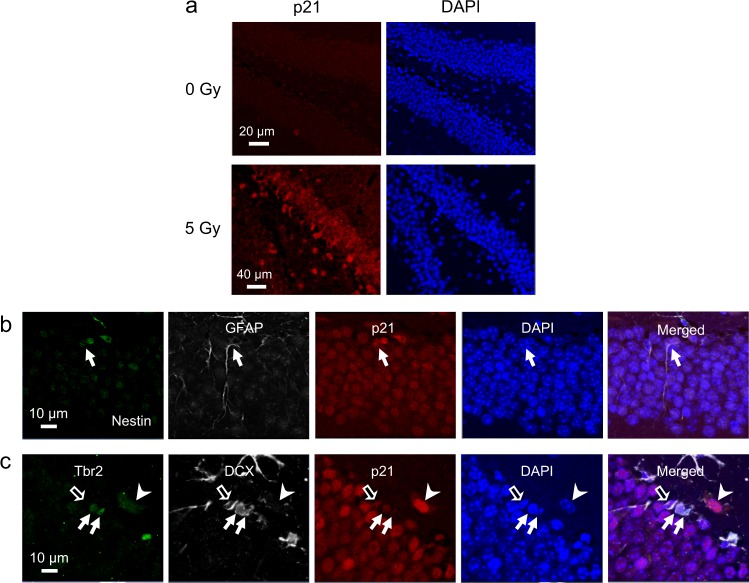
Irradiation results in upregulation of p21 in neural progenitors (NPCs) in dentate gyrus. **a** There is absence of p21 immunoreactivity in nonirradiated dentate gyrus whereas p21 nuclear immunostaining is apparent in both granule and subgranular cells at 9 weeks after 5 Gy (p21, red; DAPI, blue). **b** A type-1 cell (arrow) demonstrates p21 nuclear immunoreactivity after irradiation (nestin, green; GFAP, white; p21, red; DAPI, blue). **c** Some type-2a (Tbr2+/DCX−, arrowhead), type-2b (arrows, Tbr2+/DCX+), and type-3 neural progenitors (open arrow, Tbr2−/DCX+) demonstrate p21 nuclear immunoreactivity (Tbr2, green; DCX, white; p21, red; DAPI, blue) after irradiation

Irradiation is known to result in upregulation of p21 in mouse huppocampus^15^. In nonirradiated dentate gyrus, there was no evidence of p21 immunoreactivity. At 9 weeks after 5 Gy, we observed a generalized increase in p21 nuclear immunoreactivity in the granular cell layer of dentate gyrus (Fig. 2a). Since cells in the subgranular zone also expressed p21 immunoreactivity, sections were stained for phenotypic markers of NPCs to determine if there was differential expression of p21 in NPCs after irradiation. Among the nestin+/GFAP+ type-1 cells (Fig. 2b), 10.1 ± 2.0% demonstrated p21 nuclear immunostaining after 5 Gy, and 12.0 ± 3.8% of p21+ subganular cells were nestin+/GFAP+.

We used immunohistochemistry for T-box transcription factor (Tbr2)^16^ and DCX to further sort intermediate NPCs into type-2a (Tbr2+/DCX−), type-2b (Tbr2+/DCX+), and type-3 (Tbr2−/DCX+) cells. Of the p21+ subgranular cells, 9.2 ± 1.6% were Tbr2+, and 10.4 ± 3.7% were DCX+. Of the type-2a, type-2b, and type-3 cells, 32.5 ± 10.3%, 56.7 ± 3.3%, and 14.3 ± 6% respectively demonstrated p21 immunoreactivity after irradiation (Fig. 2c).

### Loss of p21 does not alter inhibition of newborn neurons after irradiation

**Fig. 3:**
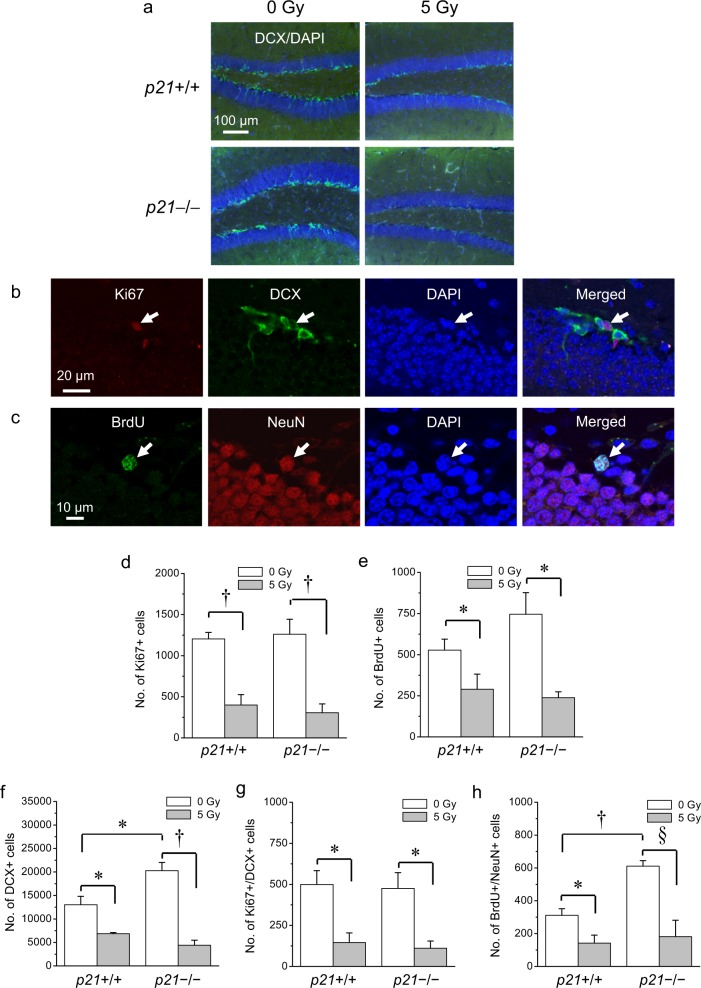
Inhibition of neurogenesis after irradiation is independent of p21. **a** Loss of DCX+ cells in dentate gyrus is apparent in both *p21*+/+mice and *p21*−/− mice at 9 weeks after irradiation (DCX, green; DAPI, blue). **b** A proliferating neuroblast (arrow) demonstrates dual Ki67/DCX staining (Ki67, red; DCX, green; DAPI, blue). **c** A newborn neuron (arrow) demonstrates dual BrdU/NeuN immunoreactivity (BrdU, green; NeuN, red; DAPI, blue). **d**, **e** At 9 weeks after 5 Gy, there is loss of Ki67+ and BrdU+ cells in dentate gyrus independent of *p21* genotype. **f**−**h** Absence of p21 results in an increase in DCX+ and BrdU+/NeuN+ cells in nonirradiated controls, but loss of DCX+ (**f**), Ki67+/DCX+ (**g**) and BrdU+/NeuN+ (**h**) cells after irradiation is independent of *p21* genotype. BrdU (50 mg/kg/day × 7 consecutive days) was given at 4 weeks and animals were killed at 9 weeks after 0 or 5 Gy. Data are represented as mean ± SEM and analyzed using two-way ANOVA, *p < 0.05, †*p* < 0.01, §*p* < 0.001, post hoc Bonferroni test with 3-4 mice per genotype per time point

To determine whether p21 plays a role in inhibition of hippocampal neurogenesis after irradiation, we compared the number of neuroblasts and newborn neurons in p21+/+ mice and p21−/− mice after 0 or 5 Gy (Fig. 3) using stereologic estimates. BrdU (50 mg/kg) was given daily for 7 consecutive days at 4 weeks after cranial irradiation. The number of DCX+ cells and BrdU-labeled NeuN+ cells were determined at 9 weeks after irradiation or at 4 weeks after the last BrdU injection. Ki67 was used as a marker for proliferating cells. Compared to controls, there was an apparent loss of DCX+ cells in both p21+/+ mice and p21−/− mice after irradiation consistent with inhibition of neurogenesis (Fig. 3a). Regardless of p21 genotype, irradiation resulted in loss of total Ki67+ cells (irradiation, *p* < 0.001; p21 genotype, *p* = not significant; two-way ANOVA; Fig. 3b, d) and BrdU+ cells (irradiation, *p* < 0.01; p21 genotype, *p* = not significant; Fig. 3c, e). Consistent with the negative effects of p21 on neurogenesis^13^, an increase in DCX+ cells and BrdU+/NeuN+ cells was observed in control nonirradiated p21−/− mice compared to wild-type mice. Irradiation resulted in loss of DCX cells (irradiation, *p* < 0.001; p21 genotype, *p* = not significant; Fig. 3f), Ki67+/DCX+ cells (irradiation, *p* < 0.01; p21 genotype, *p* = not significant; Fig. 3g), and BrdU+/NeuN+ cells (irradiation, *p* < 0.01; p21 genotype, *p* = not significant; two-way ANOVA; Fig. 3h) independent of the p21 genotype. Taken together, although p21 loss increases neurogenesis in the absence of irradiation, it does not have an effect on inhibition of neurogensis after irradiation.

### Loss of p21 does not alter loss of neural stem cells after irradiation

**Fig. 4:**
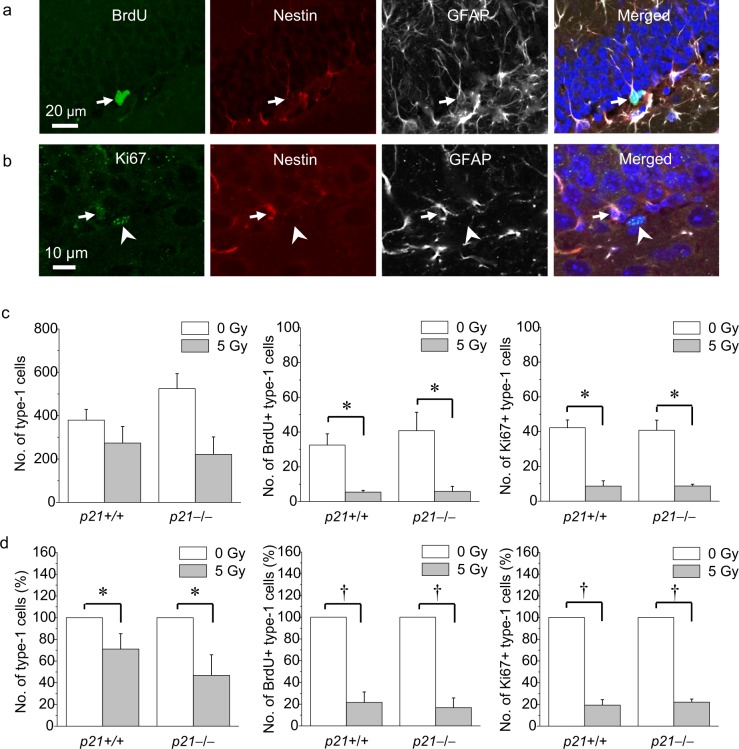
Irradiation results in depletion of proliferating and newborn type-1 cells independent of p21. **a** A newborn type-1 cell (arrow) with characteristic morphology in subgranular zone demonstrates BrdU incorporation (green), and nestin (red) and GFAP immunoreactivity (white). **b** A proliferating type-1 cell (arrow) is Ki67+ (green), nestin (red) and GFAP+ (white). An arrowhead shows a Ki67+ cell which is negative for nestin and GFAP. **c**, **d** Following a dose of 5 Gy, there is loss of newborn (BrdU+) and proliferating (Ki67+) type-1 cells independent of *p21* genotype, and the percent reduction in total, newborn and proliferating type-1 cells after 5 Gy shown in (**d**). BrdU (50 mg/kg/day × 7 consecutive days) was given at 4 weeks and animals were killed at 9 weeks after irradiation. Data are represented as mean ± SEM and analyzed using two-way ANOVA, *p < 0.05, †*p* < 0.01, post hoc Bonferroni test, with 3-4 mice per genotype per time point

We next asked if p21 loss might alter the ablation of type-1 cells after irradiation. The same 7-day BrdU paradigm was used to identify newborn type-1 cells (Fig. 4a). The number of Ki67-labeled type-1 cells (Fig. 4b) was estimated by stereology to determine the effects of p21 on proliferating type-1 cells after irradiation. In nonirradiated mice, p21 loss did not appear to alter the total number of type-1 cells, number of BrdU+ type-1 cells, and number of Ki67+ type-1 cells. A 5 Gy dose resulted in loss of BrdU+ type-1 and Ki67+ type-1 cells but the p21 genotype did not have an effect on loss of these neural stem cell populations after irradiation (BrdU+ type-1 cells: irradiation, *p* < 0.001; p21 genotype, *p* = not significant; Ki67+ type-1 cells: Ki67+ type-1 cells: irradiation, *p* < 0.0001; p21 genotype, *p* = not significant; two-way ANOVA, Fig. 4c, d).

### p21 deficiency results in differential ablation of proliferative NPCs after irradiation

**Fig. 5:**
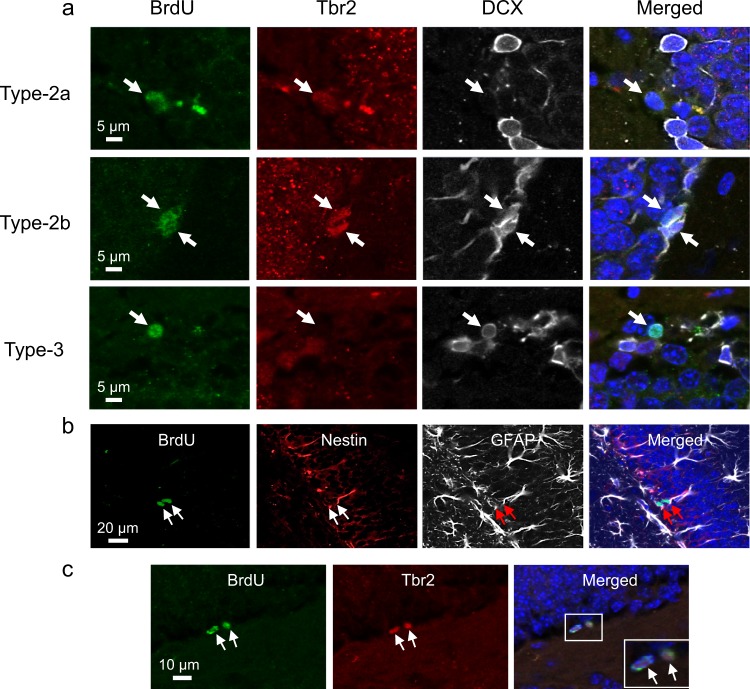
Pulsed BrdU injections label intermediate NPCs. **a** Immunohistochemistry for Tbr2 and DCX is used to sort BrdU-labeled intermediate NPCs (arrows) into type-2a (Tbr2+/DCX−), type-2b (Tbr2+/DCX+) and type-3 cells (BrdU, green; Tbr2, red; DCX, white). **b**, **c** Some BrdU+NPCs are seen as BrdU doublets, defined as two abutting BrdU+ nuclei identified on DAPI. A type-1 BrdU doublet (**b**, arrow; BrdU, green) is nestin+ (red) and GFAP+ (white), and a type-2 BrdU doublet is Tbr2+ (**c**, arrows, BrdU, green; Tbr2, red)

To determine the influence of p21 on cell fate of NPCs, mice were given a single injection of BrdU (150 mg/kg) at 4 weeks after 0 or 5 Gy, and BrdU-labeled NPC subpopulations were quantified at 2 h, 2 days, 1, and 5 weeks after BrdU. Using this BrdU paradigm, cells that demonstrated BrdU incorporation at 2 h were primarily proliferating cells. BrdU+ cells at 2 days represented a blend of proliferating and newborn cells, and those at 1 week and 5 weeks after BrdU were expected to be mostly newborn cells. Tbr2 and DCX immunohistochemistry was used to differentiate BrdU+ intermediate NPCs into type-2a (Tbr2+/DCX−), type-2b (Tbr2+/DCX+), and type-3 (Tbr2−/DCX+) cells (Fig. 5a).

**Fig. 6:**
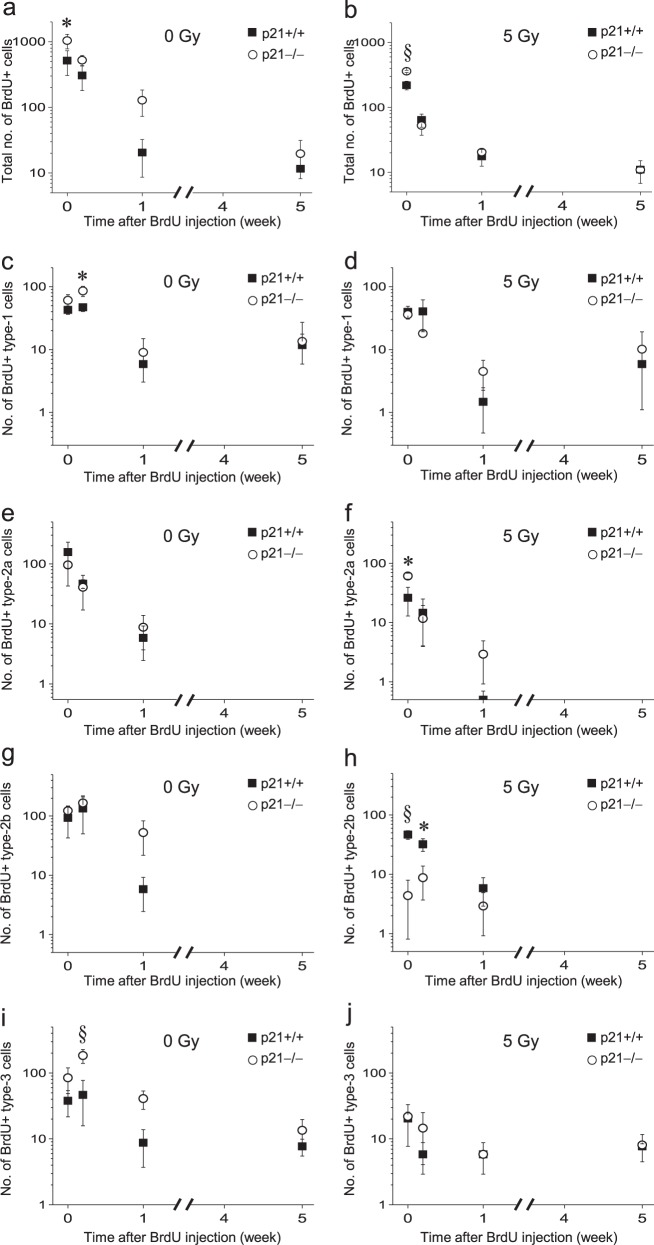
Loss of p21 results in differential ablation of NPCs after irradiation. **a** The number of BrdU+ cells in nonirradiated *p21*−/− mice is increased compared to nonirradiated *p21*+/+ mice (*p21* genotype, *p* < 0.05). **b** Loss of BrdU+ cells after irradiation is *p21* genotype dependent (*p21* genotype, *p* < 0.01). **c**, **d** For BrdU+type-1 (nestin+/GFAP+) cells, their decline over time is *p21* dependent in nonirradiated mice (**c**, *p21* genotype, *p* < 0.05) but not after irradiation (**d**). **e**, **f** The number of BrdU+type-2a (Tbr2+/DCX−) cells over time after BrdU is independent of *p21* in nonirradiated (**e**) and irradiated mice (**f**). **g**, **h** The decline in BrdU+type-2b (Tbr2+/DCX+) cells after BrdU in nonirradiated mice is independent of *p21* (**g**) whereas there is a marked loss of BrdU+ type-2b cells in *p21*−/− mice after 5 Gy compared to wild-type animals (**h**, *p21* genotype, *p* < 0.001). **i**, **j** BrdU+ type-3 (Tbr2−/DCX+) cells are increased in nonirradiated *p21*−/− mice compared to wild-type mice (**i**, *p21* genotype, *p* *<* 0.001), but their loss after irradiation (**j**) is *p21* genotype independent. A single dose of BrdU (150 mg/kg) was given at 4 weeks after 0 or 5 Gy. Cells were counted at 2 h, 2 days, 1 and 5 weeks after BrdU. Data are represented as mean ± SEM and analyzed using two-way ANOVA, *p < 0.05, †*p* < 0.01, §*p* < 0.001, post hoc Bonferroni test with 3−5 mice per genotype per time point

The number of BrdU-labeled cells declined over the 5 weeks after BrdU injection in nonirradiated mice and irradiated mice regardless of genotype (Fig. 6). In 0-Gy controls, p21 loss was associated with an increase in total BrdU+ cells (p21 genotype, *p* < 0.05; time after BrdU, *p* < 0.0001; interaction, *p* = not significant; two-way ANOVA; Fig. 6a), BrdU+ type-1 cells (p21 genotype, *p* < 0.05; time after BrdU, *p* < 0.0001; interaction, *p* = not significant; Fig. 6c) and BrdU+ type-3 cells (p21 genotype, *p* < 0.001; time after BrdU, *p* < 0.0001; interaction, *p* < 0.01; Fig. 6i), but not type-2a (p21 genotype, *p* = not significant; time after BrdU, *p* < 0.01; Fig. 6e) and type-2b cells (p21 genotype, *p* = not significant; time after BrdU, *p* < 0.01; Fig. 6g), compared to p21+/+ mice.

A dose of 5 Gy resulted in loss of total BrdU+ cells in both p21+/+ mice and p21−/− mice (p21+/+ mice: irradiation, *p* < 0.05; time after BrdU, *p* < 0.001; interaction, *p* = not significant; p21−/− mice: irradiation, *p* < 0.001; time after BrdU, *p* < 0.0001; interaction, *p* = 0.01; Fig. 6a, b and Supplementary Fig. 1a, b). Among the different NPC subpopulations in p21+/+ mice, there was loss of BrdU+ type-2a cells (irradiation, *p* < 0.05; time after BrdU, *p* < 0.05; interaction, *p* = not significant; Fig. 6e, f and Supplementary Fig. 1e) but not BrdU+ type-1 (irradiation, *p* = not significant; time after BrdU, *p* < 0.01; Fig. 6c, d and Supplementary Fig. 1c), BrdU+ type-2b (irradiation, *p* = not significant; time after BrdU, *p* = not significant; Fig. 6g, h and Supplementary Fig. 1g) and BrdU+ type-3 cells (irradiation, *p* = not significant; time after BrdU, *p* = not significant; Fig. 6i, j and Supplementary Fig. 1i) after 5 Gy.

In p21−/− mice after irradiation, there was loss of different BrdU+ NPC subpopulations, namely loss of BrdU+ type-1 cells (irradiation, *p* < 0.01; time after BrdU, *p* < 0.001; interaction, *p* < 0.05; Fig. 6c, d and Supplementary Fig. 1d), BrdU+ type-2b cells (irradiation, *p* < 0.0001, time after BrdU, *p* < 0.001; interaction, *p* = 0.01; Fig. 6g, h and Supplementary Fig. 1h) and BrdU+ type-3 cells (irradiation, *p* < 0.0001; time after BrdU, *p* < 0.0001; interaction, *p* < 0.0001; Fig. 6i, j and Supplementary Fig. 1j) but not type-2a cells (irradiation, *p* = not significant; time after BrdU, *p* < 0.05; Fig. 6e, f and Supplementary Fig. 1f). In p21+/+ mice, BrdU+ type-2b cells at 2 h and 2 days were reduced after 5 Gy to 50.0 ± 8.3% and 23.9 ± 20.3% respectively compared to controls whereas they were markedly reduced to 1.8 ± 1.8 and 4.7 ± 2.3% respectively in p21−/− mice after irradiation (Supplementary Fig. 1a-j).

After irradiation, the effect of p21 genotype demonstrated a significant independent effect on the total number of BrdU+ cells (time after BrdU, *p* < 0.0001; p21 genotype, *p* < 0.01; interaction, *p* < 0.001; Fig. 6b), and type-2b cells (time after BrdU, *p* < 0.001; p21 genotype, *p* < 0.001; interaction, *p* < 0.01; Fig. 6h), but not BrdU+ type-1 (time after BrdU, *p* < 0.05; p21 genotype, *p* = not significant; Fig. 6d), BrdU+ type-2a (time after BrdU, *p* < 0.0001; p21 genotype, *p* = not significant; Fig. 6f) or BrdU type-3 cells (time after BrdU, *p* = not significant; p21 genotype, *p* = not significant; Fig. 6j).

**Fig. 7:**
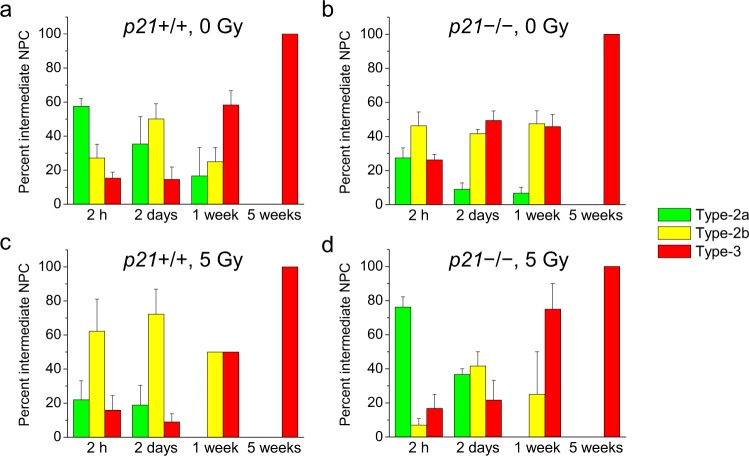
Loss of p21 alters disruption of neuronal progression over the three stages of intermediate NPCs after irradiation. **a**−**d** The distribution of BrdU+ type-2a (Tbr2+/DCX−), type-2b (Tbr2+/DCX+) and type-3 (Tbr2−/DCX+) cells as percent total intermediate NPCs (Tbr2+or DCX+) is disrupted with p21 loss and after irradiation. A single dose of BrdU (150 mg/kg) was given at 4 weeks after 0 or 5 Gy. Data are represented as mean ± SEM with 3−5 mice per genotype per time point

To further discern the influence of p21 genotype on neuronal development within the stages of transiently amplifying or intermediate NPCs after irradiation, we determined the distribution of BrdU+ type-2a, type-2b, and type-3 cells among all BrdU+/Tbr2+ and BrdU+/DCX+ cells (Fig. 7). In nonirradiated wild-type mice, the results are consistent with increasing neuronal differentiation with progression through type-2a, type-2b, and type-3 cells over the 5 weeks after BrdU (Fig. 7a). With p21 loss in nonirradiated mice, there appeared to decreased type-2a cell proliferation (p21 genotype, *p* < 0.05, time after BrdU, *p* < 0.01, interaction, *p* = not significant; Fig. 7a, b) but increased type-3 cell proliferation or enhanced differentiation into type-3 cells (p21 genotype, *p* < 0.05, time after BrdU, *p* < 0.0001, interaction, *p* < 0.01; Fig. 7a, b). After irradiation, the effect of p21 loss resulted in increased % BrdU+ type-2a cells (p21 genotype, *p* < 0.01, time after BrdU, *p* < 0.0001, interaction, *p* < 0.01) but decreased % BrdU+ type-2b cells (p21 genotype, *p* < 0.01, time after BrdU, *p* < 0.01, interaction, *p* = not significant), and % BrdU+ type-3 cells appeared unperturbed (p21 genotype, *p* = not significant, time after BrdU, *p* < 0.0001; Fig. 7c, d). These results again demonstrate the differential effects of p21 on neuronal development within the three stages of intermediate NPC development following irradiation. We were unable to detect any BrdU+/Tbr2+ cells at 5 weeks consistent with culling of type-2 cells due to death and/or differentiation over a few weeks after BrdU incorporation^12,17^.

To further assess the role of p21 on inhibition of NPC proliferation after irradiation, we determined the number of BrdU doublets, type-1 (GFAP+/nestin+) BrdU doublets (Fig. 5b) and type-2 (Tbr2+) BrdU doublets (Fig. 5c) at 2 h after BrdU. A BrdU doublet was defined as two abutting DAPI-stained nuclei that demonstrated BrdU incorporation^12^. The number of type-1 BrdU doublets increased in both nonirradiated and irradiated p21−/− mice compared to wild-type controls, and irradiation did not alter their numbers (irradiation, *p* = not significant; p21 genotype, *p* = 0.001, two-way ANOVA; Supplementary Fig. 2). In contrast, the number of type-2 BrdU doublets remained altered with p21 loss and a 5 Gy-dose reduced the number of type-2 BrdU doublets in both p21+/+ mice and p21−/− mice (irradiation, *p* < 0.001; p21 genotype, *p* = not significant).

## Discussion

Inhibition of hippocampal neurogenesis is implicated in neurocognitive decline following radiation treatment for brain tumors^18^. How DNA damage following ionizing radiation results in disruption of neuronal development remains unclear. The tumor suppressor p53 is known to regulate cellular response after DNA damage^19^. It negatively regulates proliferation and self-renewal of neural stem cells^20–22^. We recently showed that p53 loss resulted in increased ablation of neural stem cells and profound inhibition of neurogenesis in mouse dentate gyrus after irradiation despite lack of NPC apoptosis^12^. Damage of the neurovascular niche plays a role in impaired neurogenesis after irradiation^23–25^. Impaired neuronal differentiation of NPCs transplanted into the irradiated hippocampus was not altered by *p53* genotype of recipient mice^12^. Thus the effect of p53 on disruption of neuronal development after irradiation is likely to be independent of damage of the neural stem cell niche.

How p53 regulates DNA damage response in NPCs remains unclear. A well-described downstream effector of p53 is p21. In young adult mouse brain, p21 loss is associated with increased NPC proliferation but neurospheres from p*21*−/− mice demonstrated limited in vitro self-renewal^13^. It has been proposed that p21 serves to keep adult neural stem cells in relative quiescence for life-long maintenance of self-renewal. Here, p21 loss was associated with increased cell proliferation in the dentate gyrus, including enhanced proliferation of type-1 cells and neuroblasts. We also observed an increase in newborn neurons, consistent with an inhibitory effect of p21 on neurogenesis in the dentate gyrus.

Stem cells may have distinct mechanisms from progenitors to mitigate DNA damage to preserve self-renewal and differentiation^26^. Adult stem cells are known to be resistant to apoptosis after irradiation. p21 may play a role in the regulation of DNA damage response of stem cells^27^. In hematopoietic and mammary stem cells in vitro, irradiation was shown to result in activation of p21 independent of p53, and subsequent inhibition of basal activity of p53. This p21-dependent response in stem cells after DNA damage has been postulated to result in inhibition of apoptosis^28^. Neural stem cells are known to be apoptosis resistant after irradiation^14^. Here, irradiation resulted in an increase in p21 immunoreactivity in type-1 cells and intermediate NPCs. There was no evidence of acquisition of apoptosis radiosensitivity in neural stem cells with p21 loss, and we also observed no altered apoptosis radiosensitivity in proliferating type-2 cells and neuroblasts in the absence of p21. In irradiated *p21*−/− embryonic mouse cortex, there was decreased not increased apoptosis^29^. Whether these differences in p21-dependent responses post-DNA damage are related to differences in tissue-specific stem cell radiobiology, developmental or in vitro conditions, remain to be investigated.

Neural stem cells and NPCs are known to exhibit differential responses to physiologic stresses and external insults^30^. Our results here provide evidence for differential effects of p21 on NPC cell fate after irradiation. In wild-type mice, the predominant effect of irradiation appears to be death of type-2a cells and/or inhibition of their proliferation. In contrast, the major effect of irradiation with p21 loss was increased culling of proliferative type-1, type-2b, and type-3 NPCs. There is in vitro evidence that activation of p21 in certain stem cells results in induction of symmetric self-renewing divisions after irradiation^28^. Although we did not address self-renewal directly, the absence of any increase in type-1 cells at 2 days compared to at 2 h after BrdU provided no evidence to suggest induced symmetrical division of type-1 cells after irradiation. We also did not observe any p21-dependent effects on type-1 cell fate after irradiation.

Several methods including thymidine analogs, retrovirus, and transgenic animals have been used to study adult hippocampal neurogensis^1,31^. These different fate mapping approaches may contribute to the various models of NPC behavior proposed. Here we used BrdU injection paradigms to assess neuronal development. We chose a dose of 5 Gy in our study as this dose resulted in loss of about 50% of newborn neurons^12^ and was considered optimal to discern the effect of genotype on inhibition of neurogenesis after irradiation. Although our results appeared to internally consistent, no BrdU+ type-2 cells were observed at 5 weeks using the single BrdU injection paradigm. Further information may be obtained using other fate mapping methods and additional sets of phenotypic markers for NPCs.

In conclusion, there is increased hippocampal neurogenesis associated with p21 loss which may be due to enhanced NPC proliferation and/or neuronal differentiation particularly in the latter stages of intermediate NPC development. Irradiation results in inhibition of neurogenesis and loss of newborn neural stem cells. These effects appear to be independent of p21. In wild-type mice, the most pronounced effects of irradiation is death and/or inhibition of proliferation of type-2a cells. In contrast, there is increased ablation of proliferative type-1, type-2b, and type-3 NPCs after irradiation with p21 loss. Our results are thus consistent with differential effects of p21 on NPC cell fate after irradiation. Loss of p21 however does not alter the ultimate inhibition of production of new neurons after irradiation.

## Materials and methods

### Animals

C57 mice wild type (+/+) or knockout of the *p21* gene were obtained from the Jackson Laboratory (Bar Harbor, ME, USA). Mouse colonies were generated by littermate inbreeding and genotyped by PCR^12^. To avoid the potential confounding influence of sex on neurogenesis^32^, only male mice were used in the study. All animal procedures (protocol number: 18-156) were approved by the institutional animal care committee and performed according to the Canadian Council on Animal Care guidelines.

### Irradiation

Animals were irradiated at the age of 10 weeks. They were anesthetized using an intraperitoneal injection of ketamine (75 mg/kg) and xylazine (6 mg/kg) and positioned in a customized jig with the entire hippocampus irradiated using an anterior-posterior and posterior-anterior pair of 160 kV X-ray beam (Model CP160, Faxitron X-ray, Wheeling, IL, USA) defined by an 8-mm diameter lead cut-out^33^. Control (0 Gy) mice were sham-irradiated. A single dose of 5 Gy was used to evaluate the effects of the *p21* genotype on neuronal development after irradiation. Three different BrdU incorporation schedules were used for cell fate mapping as described in the Results section. BrdU was administered by intraperitoneal injection.

### Histopathology and immunohistochemistry

Under anesthesia with ketamine and xylazine, mice underwent intracardiac perfusion with 0.9% saline followed by 4% paraformaldehyde in PBS. The brain was dissected out, post-fixed for 2 days and cryoprotected in PBS with 30% sucrose. Coronal sections containing the hippocampus were cut at 40-μm thickness and stored in tissue cryoprotectant solution in 96-well plates at −20 °C prior to immunohistochemistry.

Apoptotic cells were identified based on characteristic nuclear condensation and fragmentation of apoptosis using DAPI^11,17^. The apoptotic response was further characterized using TUNEL (In Situ Cell Death Detection Kit, Roche, Indianapolis, IN, USA; Cat# 11684795910) and caspase-3 (1:1000; Cell Signaling Technology, Danvers, MA, USA; Cat# 9661) immunohistochemistry as previously described^11^.

NPCs, immature and mature neurons were identified by phenotypic markers using immunohistochemistry^11^. Primary antibodies included those against DCX (1:2000, Abcam, Cambridge, MA, USA; Cat# ab18723), GFAP (1:200, DakoCytomation, Copenhagen, Denmark; Cat# Z0334), nestin (1:200, Millipore, Billerica, MA, USA; Cat# MAB353), NeuN (1:500, Millipore; Cat# MAB377), and Tbr2 (1:200, Abcam; Cat# ab23345). Secondary antibodies were conjugated to Cy2, Cy3 (1:200; Jackson ImmunoResearch, West Grove, PA, USA; Cat# 712225150, cat#712165150) or Alexa Fluor 647 (1:200, Invitrogen, Burlington, Ontario, Canada; Cat# A31573). Colocalization of phenotypic markers with BrdU (1:200, Abcam; Cat# ab6326) or Ki67 (1:1000, Novocastra, Newcastle, UK; Cat# NCL-Ki67p) in selected sections were evaluated using a confocal laser scanning microscope (Zeiss LSM700, Carl Zeiss AG Corporate, Oberkochen, Germany). A BrdU doublet was defined as two abutting DAPI-stained nuclei that demonstrated BrdU incorporation^12^.

### Stereological analysis

Apoptotic cells and cells labeled using different phenotypic markers were counted within the dentate gyrus including a 50-µm margin of the hilus^11^. Cell counting was performed using a Zeiss Imager M1 microscope (Carl Zeiss AG Corporate) with the Stereo Investigator software (MBF Bioscience, Williston, VT, USA). The observers were blinded to the experimental groups. Details of stereology were as described previously^11^. Apoptotic cells and NPCs were counted using a counting frame and a sampling grid of 75 × 75 µm^2^ at a magnification of ×630. Every seventh section was used as the periodicity of sections sampled. For all stereology data, the coefficient of error ranged from 0.03 to 0.06.

### Statistical analysis

Cell population analysis represented data from 3 to 5 mice per genotype per dose per time point. All data represented the mean ± SEM. The significance of two independent variables such as irradiation and *p21*-genotype, or *p21*-genotype and time after BrdU on the various end-points was determined using two-way ANOVA. Pair-wise comparisons were based on post hoc analysis with Bonferroni correction for multiple comparisons. Differences were considered significant for *p* < 0.05. Statistical analyses were performed with the GraphPad Prism 5 (GraphPad Software, La Jolla, CA, USA).

## Electronic Supplementary Information

Supplementary Figure 1

Supplementary Figure 2

Supplementary Figures Caption
