# Supplementary figures and images for: Effects of p21 on adult hippocampal neuronal development after irradiation
Source: Cell Death Discov. 2018 Jul 18;4:79. doi: 10.1038/s41420-018-0081-2 (PMC6131552; doi:10.1038/s41420-018-0081-2)

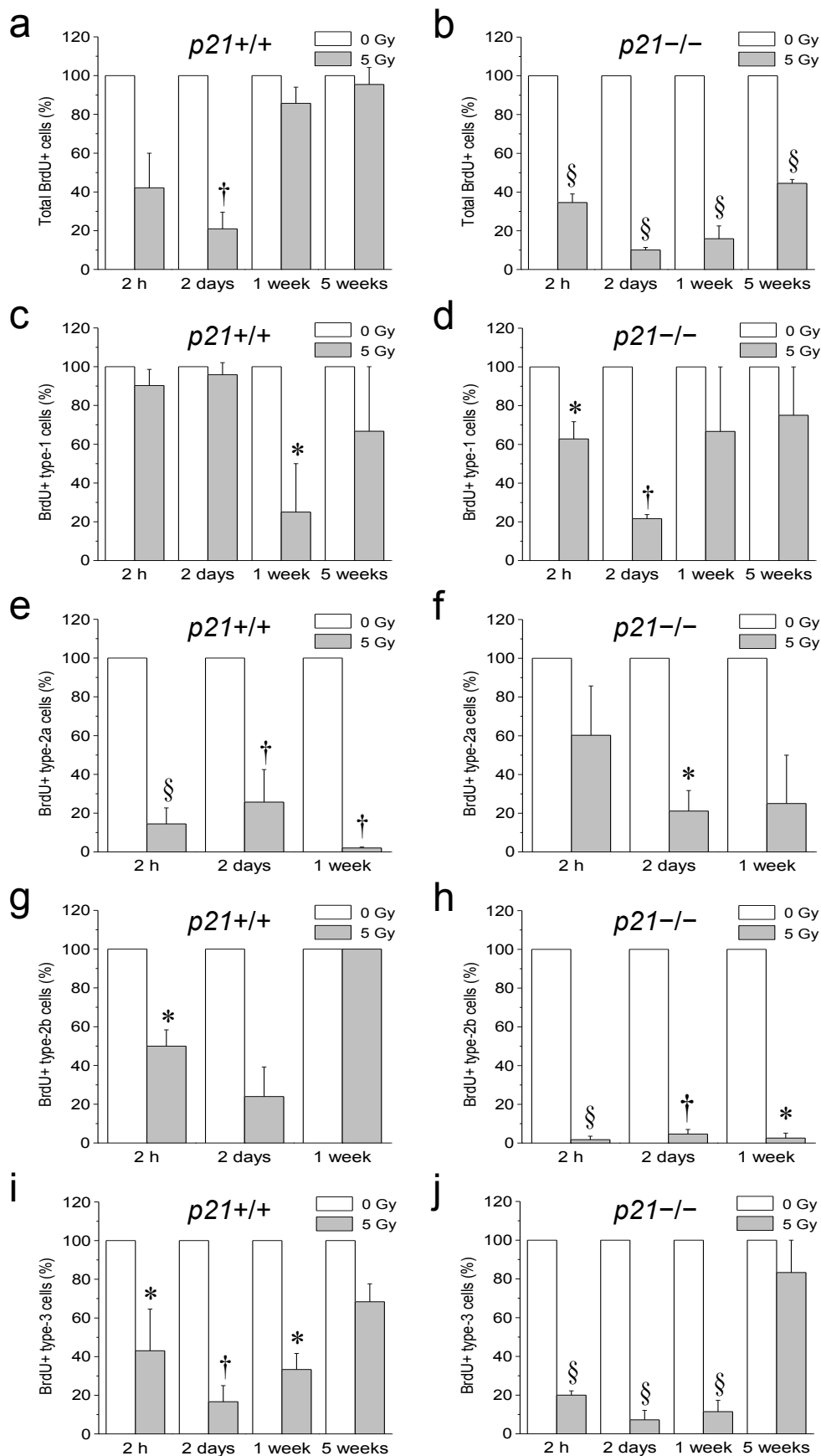

Supplementary Fig. 1

Supplement: Supplementary file 1 — Supplementary Figure 1 [file 41420_2018_81_MOESM1_ESM.pdf]

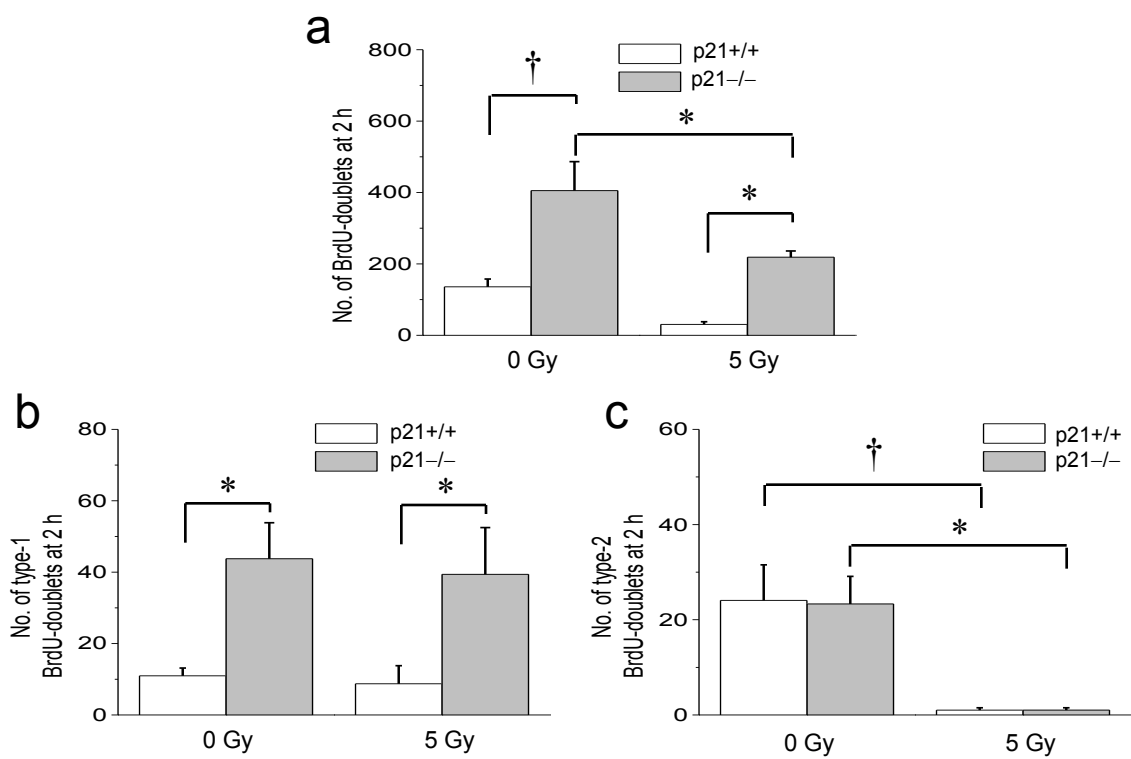

Supplementary Fig. 2

Supplement: Supplementary file 2 — Supplementary Figure 2 [file 41420_2018_81_MOESM2_ESM.pdf]
