# Supplementary material for: Effects of p21 on adult hippocampal neuronal development after irradiation
Source: Cell Death Discov. 2018 Jul 18;4:79. doi: 10.1038/s41420-018-0081-2 (PMC6131552; doi:10.1038/s41420-018-0081-2)
Supplement: Supplementary file 3 — Supplementary Figures Caption [file 41420_2018_81_MOESM3_ESM.docx]

**Supplementary Figure 1.** Lack of p21 results in differential loss of NPCs after irradiation. Decrease in BrdU labeled cells after 5 Gy is expressed as percent of cells in non-irradiated genotype controls at 2 hours, 2 days, 1 week and 5 weeks respectively after BrdU injection. BrdU (150 mg/kg) was given as a single injection at 4 weeks after 0 or 5 Gy. Data are represented as mean ± SEM, and analyzed using two-way ANOVA, **p* < 0.05, †*p* < 0.01, §*p* < 0.001, *post-hoc* Bonferroni test with 3-5 mice per genotype per time point.

**Supplementary Figure 2.** Irradiation ablates type-2 BrdU doublets but not type-1 BrdU doublets in dentate gyrus. **a** BrdU doublets are increased in non-irradiated and irradiated *p21*−/− mice compared to respective wild type mice controls (irradiation, *p* < 0.01; *p21* genotype, *p* < 0.001; interaction, *p* not significant, two-way ANOVA). **b** The number of type-1 BrdU doublets is increased in both non-irradiated and irradiated *p21*−/− mice compared to respective wild type mice controls. Irradiation dose not result in loss of type-1 doublets regardless *p21* genotype (irradiation, *p* not significant; *p21* genotype, *p* < 0.01). **c** Irradiation results in loss of type-2 BrdU doublets independent of p21 (irradiation, *p* < 0.001; *p21* genotype, *p* not significant). Data are represented as mean ± SEM, and analyzed using two-way ANOVA, **p* < 0.05, †*p* < 0.01, *post-hoc* Bonferroni test with 3-5 mice per genotype per time point.
